# Infant cortex responds to other humans from shortly after birth

**DOI:** 10.1038/srep02851

**Published:** 2013-10-04

**Authors:** Teresa Farroni, Antonio M. Chiarelli, Sarah Lloyd-Fox, Stefano Massaccesi, Arcangelo Merla, Valentina Di Gangi, Tania Mattarello, Dino Faraguna, Mark H. Johnson

**Affiliations:** 1 Dipartimento di Psicologia dello Sviluppo e della Socializzazione, Università di Padova, Padova, Italy; 2 Infrared Imaging Lab. ITAB -Institute of Advanced Biomedical Technologies and Dept. of Neuroscience and Imaging, University of Chieti-Pescara, Italy; 3 Centre for Brain and Cognitive Development, Birkbeck, University of London, United Kingdom; 4 Dipartimento di Psicologia, Università di Padova, Padova, Italy; 5 Dipartimento di Pediatria, Ospedale di Monfalcone, Monfalcone, Italy; 6 IRCCS Burlo Garofolo, Childrens Hospital Trieste, Italy

## Abstract

A significant feature of the adult human brain is its ability to selectively process information about conspecifics. Much debate has centred on whether this specialization is primarily a result of phylogenetic adaptation, or whether the brain acquires expertise in processing social stimuli as a result of its being born into an intensely social environment. Here we study the haemodynamic response in cortical areas of newborns (1–5 days old) while they passively viewed dynamic human or mechanical action videos. We observed activation selective to a dynamic face stimulus over bilateral posterior temporal cortex, but no activation in response to a moving human arm. This selective activation to the social stimulus correlated with age in hours over the first few days post partum. Thus, even very limited experience of face-to-face interaction with other humans may be sufficient to elicit social stimulus activation of relevant cortical regions.

Over the past 15 years, a large number of studies have shown remarkable consistency in identifying the brain regions that are involved in social interaction. These studies revealed the activation of a network of regions including the posterior STS at the temporal-parietal junction, the temporal poles and the dorsal medial prefrontal cortex, a network sometimes referred to as “the social brain”^1^. While there is agreement about the structures that compose this network in adults, much controversy surrounds the ontogenetic factors that result in this human adult specialisation. More specifically, current active debates in cognitive neuroscience concern whether certain regions of the cortex are genetically specified “modules” for social perception^2^, or whether the functions of these regions arise largely as a product of acquired expertise^3^ (see also^4^). Direct evidence from human newborns has, to date, not been available due to the technical challenges of functional brain imaging with awake alert babies.

We have previously shown, using functional near infrared spectroscopy (fNIRS), that five-month-old infants show bilateral activation of posterior temporal cortex sites in response to social video clips, a pattern that is not seen in response to static or dynamic non-social scenes (vehicles, machinery etc.)^5^. The findings in five month-olds correspond well with functional MRI studies in adults of the activation of the posterior superior temporal sulcus^6^,^7^, suggesting precocial functional specialization of some parts of the social brain network. While these previous results indicate rapid development of parts of the social brain, they cannot resolve the long-standing issue of whether or not these regions are functional prior to relevant sensory experience, since by five months of age most babies have engaged in hundreds of hours of face-to-face contact with other humans. By running the same experiment with one to four day old newborns, who, at most, have only a few hours of experience of face-to-face interaction, we can address the role of this postnatal experience on social brain functions more directly. Furthermore, using a second stimulus like a human arm manipulating an object, we can investigate possible early differences in processing between social stimuli (relevant to interaction with other humans) as opposed to biological motion per se.

Predictions for the active areas of the arrays were informed by the spatial resolution of fNIRS [light transport models^8^; structural infant MRI^9^] and from the findings of previous work on human action processing in infants^10^,^5^. The channel separation used in the current study was predicted to have a mean depth sensitivity of approximately 1 cm from the skin surface in newborn infants, potentially allowing measurement of both the gyri and parts of the sulci near to the surface of the cortex. The fNIRS headgear was aligned to scalp and anatomical landmarks, consistent with the 10–20 electrode placement system^11^, so that general comparisons could be made with adult findings from previous fMRI research. The probes covered inferior frontal and temporal regions of each hemisphere (Figure 1).

In accord with the findings discussed above, we predicted that if infants were engaging the same neural mechanisms for processing social stimuli as adults then activation in the current study would be localised to the posterior-temporal region of the probes for the dynamic social stimuli, but not for the dynamic non-social mechanical stimuli (Experiment 1). However, if the posterior STS region is not yet specialized for dynamic social stimuli in newborns, then no difference in activation between the two experimental conditions will be observed. In Experiment 2 we sought to determine whether activation patterns observed in response to the social stimuli in Experiment 1 are also observed when newborns view another type of biological motion.

## Results

**Figure 1:**
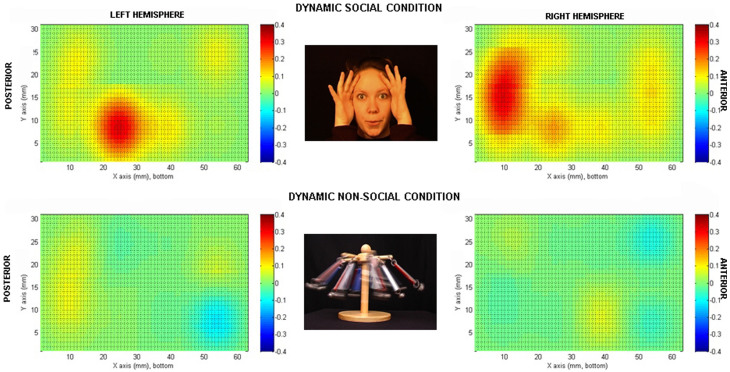
Re-constructed topographic images of the increase in HBO_2_ concentration (microM) at 7 seconds post stimulus onset for each hemisphere. Bilateral activation is evident for the H-F stimuli in the TO area. The activation is not evident for the M stimuli. The images correspond to the set of channels for each hemisphere.

**Figure 2:**
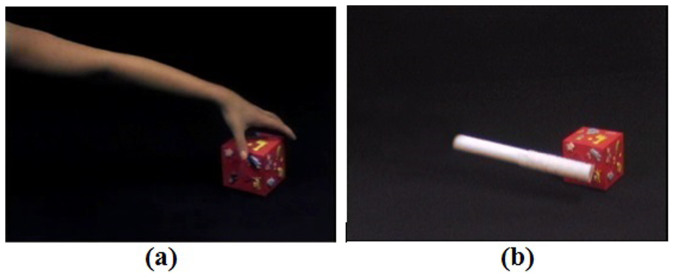
Stimuli used in Experiment 2. *2a* representing the H-A stimulus and *2b* the M stimulus.

**Figure 3:**
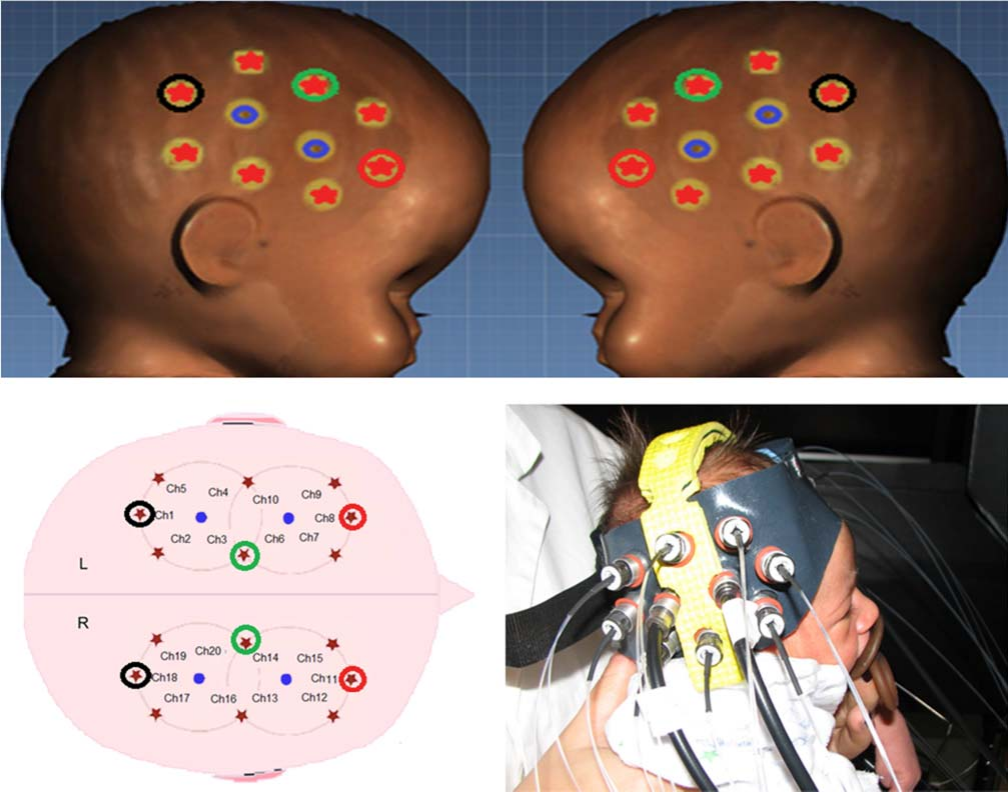
3D reconstructed infant head (cranial circumference: 34.5 cm) (Facescan, Breuckmann) and layout of the optodes and channels within the fNIRS probes. There were 10 channels for each hemisphere, with source (red stars) - detector (blue circles) separations of 1.8 cm. An infant wearing the fNIRS probe and headgear (*see also* Figure S1 and S2).

Seventeen newborns were recruited in Experiment 1 and 11 newborns in Experiment 2. Two newborns were disregarded from data analysis in each experiment for technical problems during the experiment (analysis was computed on 15 children). Informed consent was obtained from the parents of all the newborns tested. Newborns (postnatal age 24–120 hours) were shown two experimental conditions (Figure 1 and Figure 2) of visual and naturalistic dynamic social stimuli: naturalistic dynamic face stimuli (H-F) such as ‘Peek-a-boo' hand games and eye movements in Experiment 1 and a naturalistic dynamic arm stimuli (H-A), in Experiment 2. In both experiments the human stimuli where compared with dynamic non-biological mechanical stimuli (M) (moving cog wheels and pistons in Experiment 1, and a stick attaching and moving a toy like a red cube in Experiment 2), and a baseline condition consisting of naturalistic static non-biological stimuli (B; colour photographs of vehicles). The NIRS headgear was placed on the infant's head (Figure 3) and changes in oxy-haemoglobin (HbO_2_) and deoxy-haemoglobin (HHb) concentration (μmol) were calculated relative to baseline and used as haemodynamic indicators of neural activity. In terms of their behaviour, newborns were equally attentive to both types of stimuli in both experiments. We observed no significant difference in average looking time (8.9 sec for the H-F stimuli and 8.5 sec for the M stimuli in Experiment 1 and 8.4 sec for the H-A stimuli and 8.2 sec for the M stimuli in Experiment 2). Furthermore, the maximum number of trials per condition and per participants that were rejected on the basis of inattentiveness (looking away from the screen for an accumulated total of more than 5 sec while the stimuli were on) were again similar in both experiments (10 for the H-F stimuli and 12 for the M stimuli in Experiment 1 and 15 for the H-A stimuli and 17 for the M stimuli in Experiment 2, with a minimum of 18 good trials for both the H-F and M stimuli in Experiment 1, and 17 good trials for again both the H-A and M stimuli in Experiment 2).

### Experiment 1

The statistical analysis of HbO_2_ signals and beta values using uncorrected t-tests showed significant activation to the H-F relative to baseline stimuli in channel 4 (t = 2.74, df = 14, p = 0.016, uncorrected) and channel 18 (t = 3.75, df = 14, p = 0.002, uncorrected), as can be seen in the reconstructed topographic images (Figure 1). Bonferroni correction revealed that only channel 18 reached significance (p = 0.04, corrected). No channels showed significant activation/deactivation to the M stimuli. For channel 18, activation to H-F resulted in higher responses than for M (t = 3.0, df = 14, p = 0.005). No significant HHb changes were found. For the averaged haemodynamic time courses for the change in concentration of HbO_2_ and HHb in response to the H-F and M stimuli for all channels see the supplementary materials (Figure S1 and S2). In line with previous studies^1^,^12^,^13^, the increase in HbO_2_ concentration during H-F reached a maximum value towards the end of each trial, at 7 to 8 seconds post stimulus onset.

**Figure 4:**
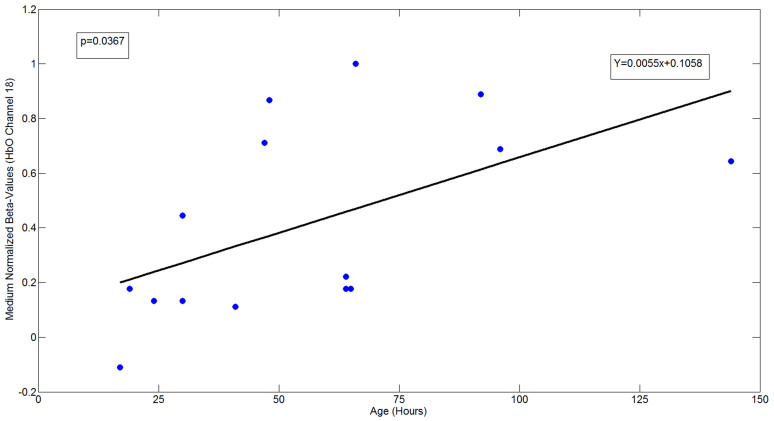
Linear regression of the normalized beta-values of HbO_2_ concentration changes within channel 18 as a function of age (hours from the time of birth). The regressions show an effect of the age (ch18: p = 0.037) on the intensity of the activation.

Secondary analysis comparisons were made for each experimental condition to investigate the effect of postnatal age within the group. This analysis was conducted on channel 4 and 18, given that the significant response in these channels is in a similar region to that found in the five month old infants^5^. Linear regression of normalized beta-values of HbO_2_ changes within channel 4 and 18 was conducted as a function of age (hours from the time of birth). The regression shows that there was a significant effect of age (only in ch18: p = 0.037 positive related) on the degree of activation (*see* Figure 4) in response to the stimulus. There were no significant regression effects within these channels for the M stimuli, nor there was any association with delivery method (i.e. caesarean versus spontaneous labour). Thus, there is no evidence that the different types and quantities of drugs administered to mothers during the two kinds of deliveries influenced newborns'states of alertness, behaviour, activity or hemodynamic response at the point of testing. Furthermore, the effect was not evident when gestational age was considered, indicating that very early postnatal experience is an important factor.

### Experiment 2

The statistical analysis of HbO_2_ signals and beta values using uncorrected t-tests show no significant effects in any channels to the arm condition and, furthermore, no channels showed significant activation/deactivation to the M stimuli. No significant HHb changes were found (Figure 2). A post hoc power analysis revealed that on the basis of the mean and SD of the absolute measures of Haemoglobin across all valid channels, an n of approximately 8 would be needed to obtain statistical power at the recommended .80 level (d = .46; Cohen, 1988). A similar effect size was found for Experiment 1 (d = 0.43).

## Discussion

Our results are consistent with the selective activation of some parts of the cortical social brain from shortly after birth, and thus provide evidence for functionality prior to significant postnatal sensory experience. This cortical activation was observed while infants viewed a dynamic face but not a moving arm. One reason for this is that the action observed by the newborn may need to contain communicative cues (such a face with direct gaze). However, several factors need to be considered before strong conclusions can be drawn.

First, as stated above we observed a significant correlation between the age (in hours) of our newborns, and the strength of their activation in response to the social stimuli. No such correlation was found with the other stimuli. This indicates that brief experience of face-to-face interaction with caregivers may be required in order to induce this specific cortical activation. Further research will be required to establish whether the experience received needs to be specific, or whether general exposure to patterned light or other factors is sufficient to elicit the activation, as has been observed in some other species^14^,^15^. In addition, it needs to be borne in mind that to draw a line for the onset of the role of experience at birth is very difficult, particularly given that the target of interest in this study, the superior temporal sulcus region, is known to be multimodal in its responses [e.g.^16^]. Another factor worthy of consideration is suggested by a recent work^17^. Since the superior temporal sulcus is known to respond to multiple modalities, it is possible that relevant experiences could occur in the womb, particularly given that we already know infants learn from voices prior to birth. Importantly, however, the visual stimuli in the current experiments were not accompanied by sound, and so the possibility that seeing human action is directly associated with the human voice heard while in the womb remains highly speculative. Nevertheless, our result is consistent with the very rapid development of primate visual acuity and face processing after treatment for congenital cataract^18^,^19^ or after early face stimulus deprivation^20^.

While there is a substantive literature indicating that human newborns preferentially orient toward faces and face-like stimuli [*see*^21^ *for review*], it does not necessarily follow that this well-studied behaviour is mediated by the same cortical structures identified in the present study. In primates, attention toward other group members and the objects of their attention is mediated by neural circuits that transduce sensory information about others and translate that information into value signals that bias orienting. This process likely proceeds via two distinct but integrated pathways: an ancestral, sub-cortical route that mediates crude but fast orienting to animate objects and faces; and a more derived route involving cortical orienting circuits that mediate nuanced and context-dependent social attention^22^. Converging evidence indicates that initial orienting to faces in human newborns is controlled by a sub-cortical pathway^21^, and types of saccades supported by cortical circuitry in adults are not always present in young infants^23^. Thus, while we have demonstrated with the current results that foveation of a stimulus results in specific cortical activation, it remains unknown whether or not this activation directs the initial selective orienting behaviour of newborns.

In comparing the findings from Experiment 1 to those of a similar study conducted with older infants, we conclude that the selective activation observed in newborns is in a highly similar location to the posterior superior temporal region activation seen to the identical human stimuli in previous work with five month olds^5^. However, the inferior frontal activation we reported in five month old infants cannot be assessed in the newborns, as the channel layout was different in the current study. Future work will be required to investigate the localization of these effects further.

Finally, since non-human biological motion stimuli have not been presented to the newborns, our results do not answer the question of whether and when in development information processing becomes tuned to conspecifics [*see*^24^]. In addition, and as with other most other studies involving social stimuli like faces^21^, future work could further assess the importance of low level psychophysical characteristics of faces or direct eye gaze (e.g., black-white contrast; optimal spatial frequencies, etc.). For example, an early preference for direct gaze may be strengthened by the high-contrast and spatial frequency of these stimuli^21^, but, nevertheless, lead to subsequent specialization of cortical areas for social stimuli more broadly. Whatever the exact neural and psychophysical basis of social orienting in newborns, the selective cortical activation we have demonstrated informs key debates in social cognitive neuroscience, and is of high potential relevance to future studies of newborns at-risk for later emerging disorders of social cognition such as autism, in which such specialisation is delayed or atypical^31^.

## Methods

### Experimental procedure

The study was conducted at the Pediatric Unit of the Hospital of Monfalcone. For Experiment 1 fifteen newborns (postnatal age 24–120 hours; mean 56 hours; 11 spontaneous labour and 4 caesarean section) were recruited for the study through the Pediatric Unit of the Hospital of Monfalcone. A further two infants were excluded due to technical problems with data collection. For Experiment 2, ten newborns (postnatal age 24–120 hours; mean 56 hours; 7 spontaneous labour and 3 caesarean were recruited. A further two infants were recruited but were excluded from the study because of fussiness (n = 1) or they had noisy data (n = 1). All infants had normal birth weight (>2,400 g) and Apgar index scores (between 8 and 10 at the fifth minute after birth). The recruitment of participants and the entire procedure was approved by the Ethical Commettee of Psychology research (University of Padua). Written informed consents were obtained from all participants' parents.

The infants sat on an experimenter's lap and were encouraged to watch the stimuli displayed on a 26-inch screen with a viewing distance of approximately 30 cm. The visual stimulation paradigm was adopted from a previous fNIRS study with five-month-old infants^5^. In Experiment 1, the conditions consisted of: full colour approximately life-size dynamic social video clips - H-F (dynamic face) - of female actors who either moved their eyes left or right, their mouth in silent vowel movements, or performed hand games; ‘Peek-a-boo' and ‘Incy Wincy spider', and dynamic non-social video clips (M) of machine cogs and pistons and moving mechanical toys (Figure 1). These stimuli were selected because they involved complex interacting curvilinear motion patterns that served as a good control for complex manual and facial motion.

In Experiment 2, a human action stimulus was again used, but rather than a face, the video contained an arm. During the condition, the arm entered the scene, reached for, and grasped an object (H-A dynamic arm, *see* Figure 2). This condition was compared with a non-human version (M) with a mechanical tool grasping the object rather than an arm.

In H-A the hand grasps the target on the surface with its natural fluid motion; whereas the mechanical tool (M) was a rod attaching to the target through a Velcro strip, moving in a rigid and linear way.

The procedure and baseline trials were identical to Experiment 1, although the two mechanical conditions (M) were different across the two experiments since we wanted to further control whether the dynamic action made by an arm (H-A) is different from activation produced by the same action but made by a mechanical tool (M).

The stimulation paradigm continued on a cyclical loop of B-H (F or A)-B-M presentation (10 seconds for each condition, 40 seconds for each trial) until the infants became tired or fussy (the average length of the presentation was three minutes). The baseline condition - B - consisted of full-colour static images of different types of transport (i.e. cars, helicopters) presented randomly for 10 seconds with each image presented for a pseudo-random duration (1–3 seconds). These images were selected to be colourful, complex and interesting, and ensured that newborns remained attentive to the screen. The overall surface area of the displayed experimental stimuli and baseline stimuli were equivalent.

Note that fNIRS studies with adults use a blank screen as the baseline, but this is not possible when working with infants, therefore the static images act as the baseline for the activated experimental period containing the human action video clips.

### Data acquisition and instrumentation

To investigate cortical activation, fNIRS measurements were made using a commercial frequency-domain oximeter (Imagent, ISS Inc.). The Imagent system is equipped with 16 sources (32 laser sources, 16 at 690 nm, 16 at 830 nm) and 4 photomultiplier tube detectors. The source light of the system is modulated at 110 MHz while the detectors are modulated at 110 MHz plus 5 kHz for heterodyne detection. The lasers of each source are time-multiplexed during measures. The light power emitted by the lasers at the fibre end is <4 mW/cm^2^, within the ANSI standard limits and permitting safe measurements. The sample rate was set to 16 Hz in order to obtain a good signal to noise ratio. DC and AC attenuations and phase shifts of the modulated light through the tissue were recorded. The optical source fibre and detection bundles were arranged in two flexible rubber probes, which were fixed to the head through a custom-built helmet. Source-detector distance was set to 1.8 cm, and 10 channels (couple source-detector) were configured for each hemisphere (Figure 3). The set source-detector separation is adequate for the population investigated^25^,^26^,^27^. Before the infants' began the study, measurements of their head circumference, and distance between glabella, ears and inion were taken and the location of the channels and probes relative to these anatomical landmarks were recorded. Each probe was placed on the temporal region, with the midpoint at a fixed distance of 6 cm from the centre of the forehead, aligned approximately with T3/T4 of the 10–20 system on the average newborns head (17 infants; median cranial circumference: 34.5 ± 1.4 cm range). The standard deviation of the maximum displacement (half head circumference) among newborns, measured from the centre of the forehead assumed as reference point, was estimated as low as 0.7 cm. The posterior half of each probe lies approximately over the scalp locations T5/T6, analogous to the region of interest^4^.

Since the inter-fibre distance was larger than the standard deviation of cranial circumference, the data from the same channel among different children could be considered as coming from similar cortical areas.

### Data processing and analysis

Fast Fourier Transform of the signal was performed to compute the AC component (amplitude), DC component (average Continuous Wave “CW” intensity) and phase shift of the waveform signal from the 20 channels. The concentration changes were assessed using the modified Lambert Beer law^28^ with an estimated Differential Pathlength Factors equal to 4.67 (830 nm) and 5.13 (690 nm). The data were band-pass filtered (0.04–0.5 Hz, FIR digital filter) to attenuate slow drifts and high frequency noise, mainly caused by physiological noise such as breathing and heart pulse. Movement artifacts were identified on and removed from the time course of each channel' signal by means of a semi-automated procedure and linear interpolation^29^,^30^,^13^. A total of 22 trials out of 75 trials were corrected for movement artifacts. For each infant we included data from 4 to 7 (mean 5) full responses to each B-H/F or H/A-B-M sequence. The neural activity in response to the H/F in Experiment 1 or H/A in Experiment 2 and M stimuli respect to B stimuli was modelled as a square-wave function lasting 10 seconds. This covariate was convolved with a hemodynamic response function (HRF) and compared to the filtered HbO_2_ concentration changes to yield appropriate predictors (beta values). This was done in a generalized linear model (GLM) and in analogy with fMRI analysis.

For the analyses, we used an adult HRF. The mean square deviation between the model and filtered HbO_2_ concentration changes was evaluated. In order to remove trials affected by motion artifacts, on the measurements collected from each participant, trials with channels that showed deviation within the population ≥ 99% percentile were disregarded. The rejection rate for stimuli was 7% on average, with a maximum of two disregarded responses of the same stimulus in one newborn. Statistical analysis was performed on beta values of HbO_2_ concentration changes. We evaluated the average response for each channel weighted on the number of received stimulus trials for each infant. A t-test analysis evaluated statistical significance of the average activation/deactivation within each channel (activation/deactivation defined as a significant increase/decrease in HbO_2_).

## Author Contributions

T.F. planned, run and collected the data, and wrote the paper. A.C. analysed the data and wrote the methodology. S.L.F. helped planning the experiments and reviewed the manuscript. S.M. prepared the stimuli, built the probe and gave the technical support during the testing; reviewed the manuscript. A.M. analysed the data and reviewed the manuscript. V.D.G. tested the newborns and reviewed the manuscript. T.M. helped testing the newborns. D.F. gave the medical support during the testing, discussed the results and reviewed the manuscript. M.H.J. advised on planning and interpretation, and co-wrote the paper.

## Supplementary Material

Supplementary Information Average time course changes in HbO2 and HHb concentration (µM)
