# Supplementary material for: Infant cortex responds to other humans from shortly after birth
Source: Sci Rep. 2013 Oct 4;3:2851. doi: 10.1038/srep02851 (PMC3790196; doi:10.1038/srep02851)
Supplement: Supplementary Information — Average time course changes in HbO2 and HHb concentration (µM) [file srep02851-s1.pdf]

Infant cortex responds to other humans from shortly after birth

Teresa Farroni[1,3], Antonio Chiarelli[2], Sarah Lloyd-Fox[3], Stefano Massaccesi[4], Arcangelo Merla[2], Valentina Di Gangi[1], Tania Mattarello[1], Dino Faraguna[5,6], Mark H. Johnson[3].

[1] *Dipartimento di Psicologia dello Sviluppo e della Socializzazione, Università di Padova, Padova, Italy,*
[2] *Infrared Imaging Lab. ITAB -Institute of Advanced Biomedical Technologies and Dept. of Neuroscience and Imaging, University of Chieti-Pescara, Italy,*
[3] *Centre for Brain and Cognitive Development, Birkbeck, University of London, United Kingdom,*
[4] *Dipartimento di Psicologia, Università di Padova, Padova, Italy,*
[5] *Dipartimento di Pediatria, Ospedale di Monfalcone, Monfalcone, Italy*
[6] *IRCCS Burlo Garofolo, Childrens Hospital Trieste, Italy*

Figure S1

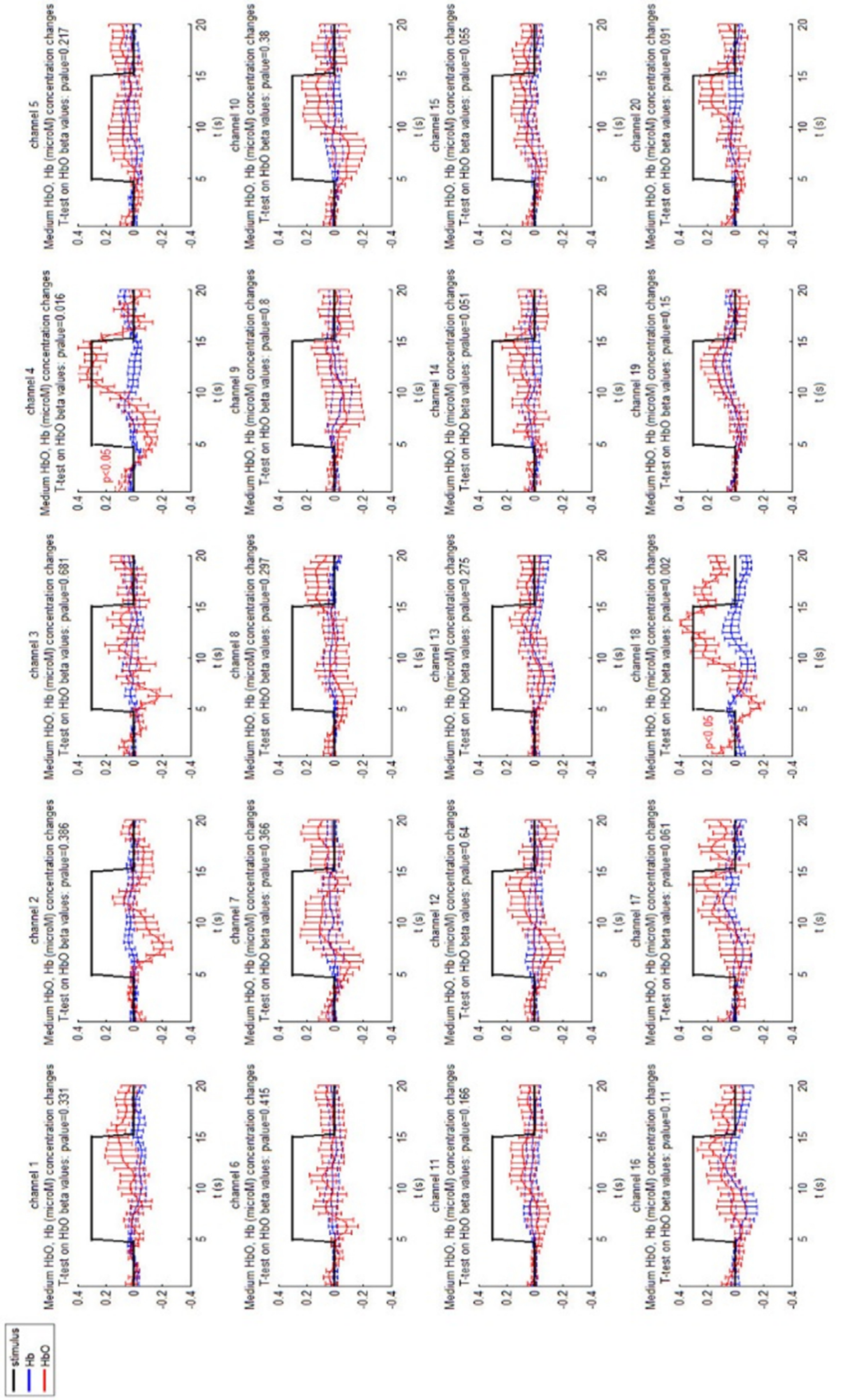
stimulus
Hb
HbO
channel 1
Medium HbO, Hb (microM) concentration changes
T-test on HbO beta values: pvalue=0.331
channel 2
Medium HbO, Hb (microM) concentration changes
T-test on HbO beta values: pvalue=0.386
channel 3
Medium HbO, Hb (microM) concentration changes
T-test on HbO beta values: pvalue=0.581
channel 4
Medium HbO, Hb (microM) concentration changes
T-test on HbO beta values: pvalue=0.016
p<0.05
channel 5
Medium HbO, Hb (microM) concentration changes
T-test on HbO beta values: pvalue=0.217
channel 6
Medium HbO, Hb (microM) concentration changes
T-test on HbO beta values: pvalue=0.415
channel 7
Medium HbO, Hb (microM) concentration changes
T-test on HbO beta values: pvalue=0.366
channel 8
Medium HbO, Hb (microM) concentration changes
T-test on HbO beta values: pvalue=0.297
channel 9
Medium HbO, Hb (microM) concentration changes
T-test on HbO beta values: pvalue=0.8
channel 10
Medium HbO, Hb (microM) concentration changes
T-test on HbO beta values: pvalue=0.38
channel 11
Medium HbO, Hb (microM) concentration changes
T-test on HbO beta values: pvalue=0.166
channel 12
Medium HbO, Hb (microM) concentration changes
T-test on HbO beta values: pvalue=0.64
channel 13
Medium HbO, Hb (microM) concentration changes
T-test on HbO beta values: pvalue=0.275
channel 14
Medium HbO, Hb (microM) concentration changes
T-test on HbO beta values: pvalue=0.051
channel 15
Medium HbO, Hb (microM) concentration changes
T-test on HbO beta values: pvalue=0.055
channel 16
Medium HbO, Hb (microM) concentration changes
T-test on HbO beta values: pvalue=0.11
channel 17
Medium HbO, Hb (microM) concentration changes
T-test on HbO beta values: pvalue=0.061
channel 18
Medium HbO, Hb (microM) concentration changes
T-test on HbO beta values: pvalue=0.002
p<0.05
channel 19
Medium HbO, Hb (microM) concentration changes
T-test on HbO beta values: pvalue=0.15
channel 20
Medium HbO, Hb (microM) concentration changes
T-test on HbO beta values: pvalue=0.091

Figure S2

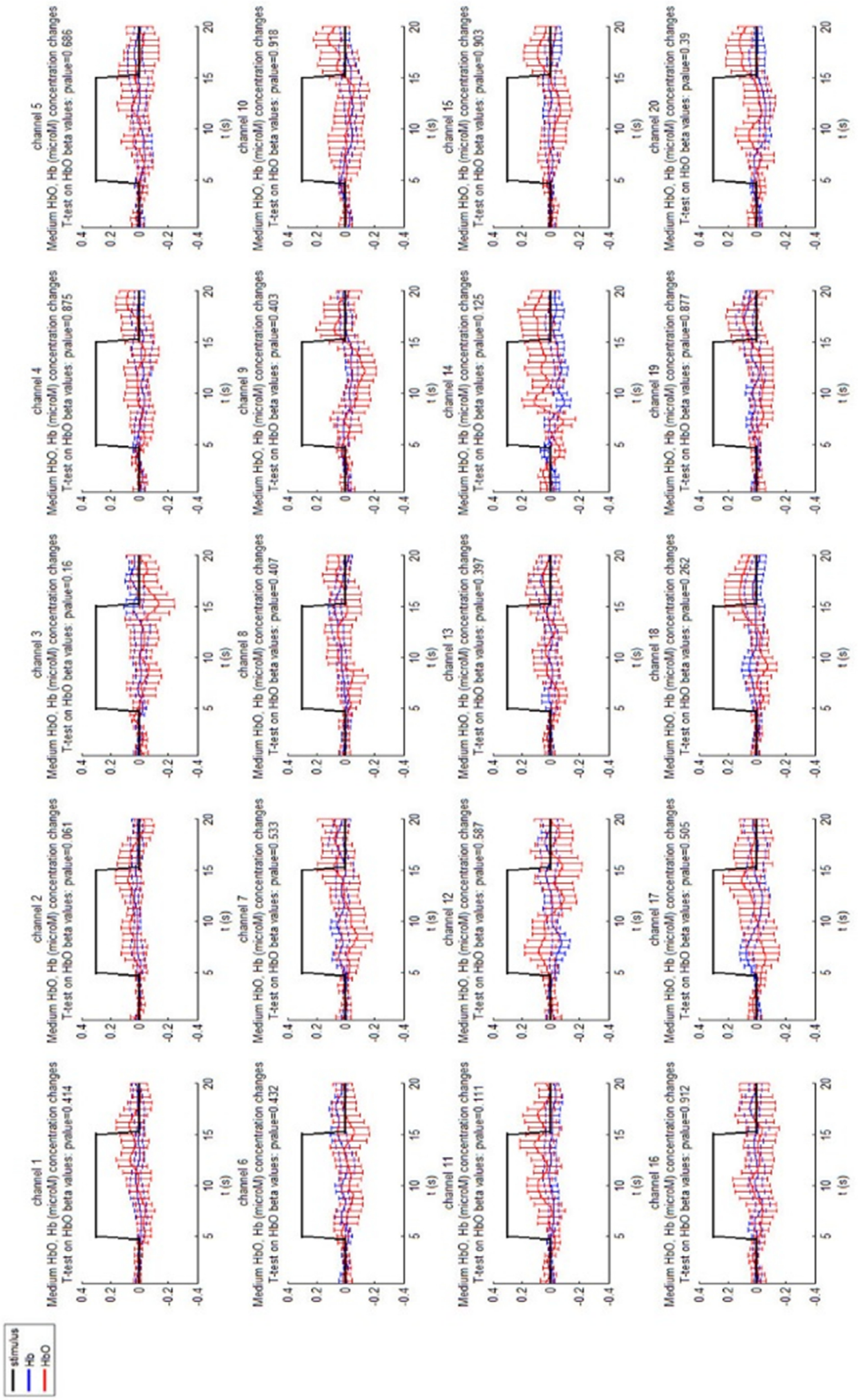
stimulus
Hb
HbO
channel 1
Medium HbO, Hb (microM) concentration changes
T-test on HbO beta values: pvalue=0.414
channel 2
Medium HbO, Hb (microM) concentration changes
T-test on HbO beta values: pvalue=0.061
channel 3
Medium HbO, Hb (microM) concentration changes
T-test on HbO beta values: pvalue=0.16
channel 4
Medium HbO, Hb (microM) concentration changes
T-test on HbO beta values: pvalue=0.875
channel 5
Medium HbO, Hb (microM) concentration changes
T-test on HbO beta values: pvalue=0.686
channel 6
Medium HbO, Hb (microM) concentration changes
T-test on HbO beta values: pvalue=0.432
channel 7
Medium HbO, Hb (microM) concentration changes
T-test on HbO beta values: pvalue=0.533
channel 8
Medium HbO, Hb (microM) concentration changes
T-test on HbO beta values: pvalue=0.407
channel 9
Medium HbO, Hb (microM) concentration changes
T-test on HbO beta values: pvalue=0.403
channel 10
Medium HbO, Hb (microM) concentration changes
T-test on HbO beta values: pvalue=0.918
channel 11
Medium HbO, Hb (microM) concentration changes
T-test on HbO beta values: pvalue=0.111
channel 12
Medium HbO, Hb (microM) concentration changes
T-test on HbO beta values: pvalue=0.587
channel 13
Medium HbO, Hb (microM) concentration changes
T-test on HbO beta values: pvalue=0.397
channel 14
Medium HbO, Hb (microM) concentration changes
T-test on HbO beta values: pvalue=0.125
channel 15
Medium HbO, Hb (microM) concentration changes
T-test on HbO beta values: pvalue=0.903
channel 16
Medium HbO, Hb (microM) concentration changes
T-test on HbO beta values: pvalue=0.912
channel 17
Medium HbO, Hb (microM) concentration changes
T-test on HbO beta values: pvalue=0.505
channel 18
Medium HbO, Hb (microM) concentration changes
T-test on HbO beta values: pvalue=0.262
channel 19
Medium HbO, Hb (microM) concentration changes
T-test on HbO beta values: pvalue=0.877
channel 20
Medium HbO, Hb (microM) concentration changes
T-test on HbO beta values: pvalue=0.39
t (s)

Figure S3

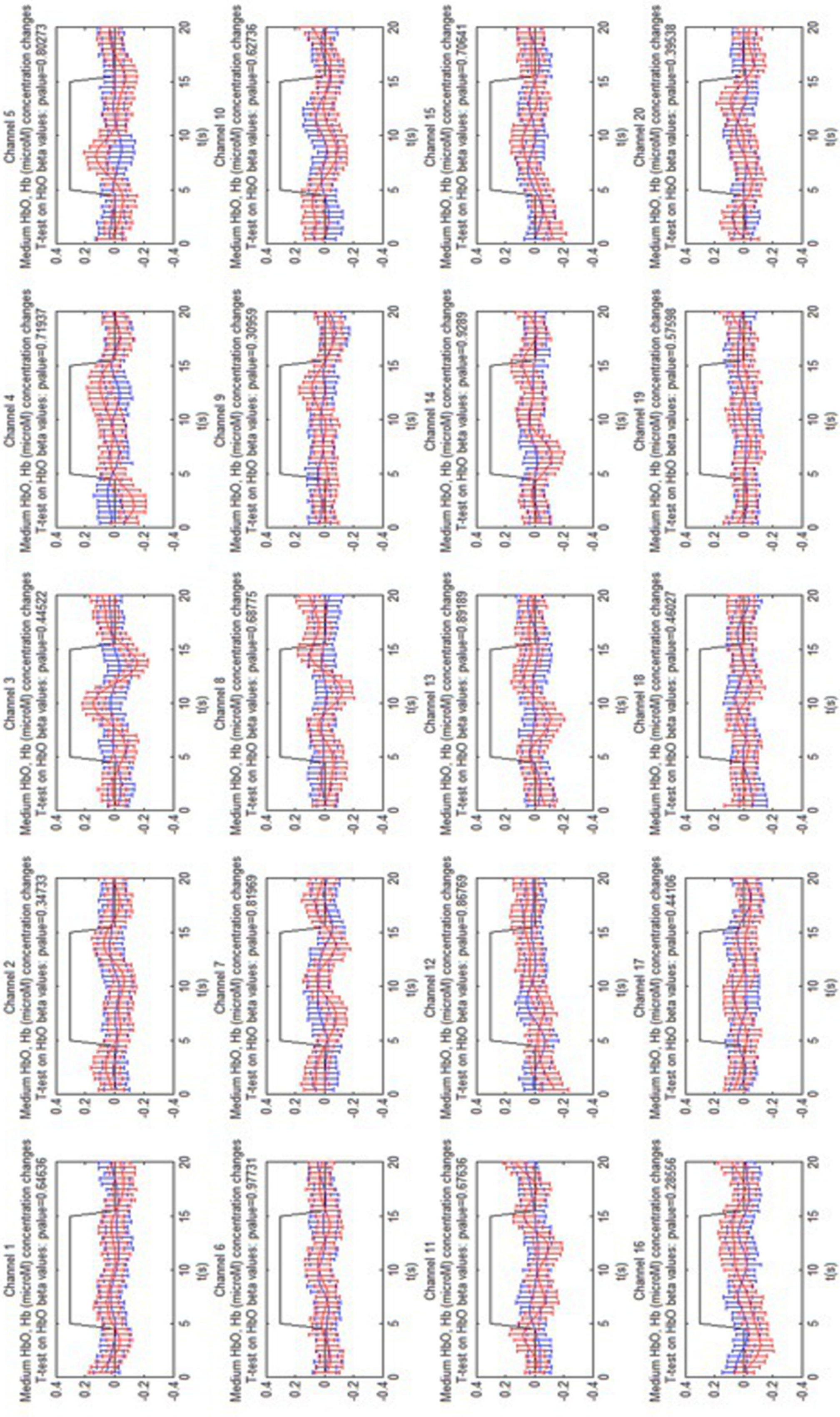

Figure S4

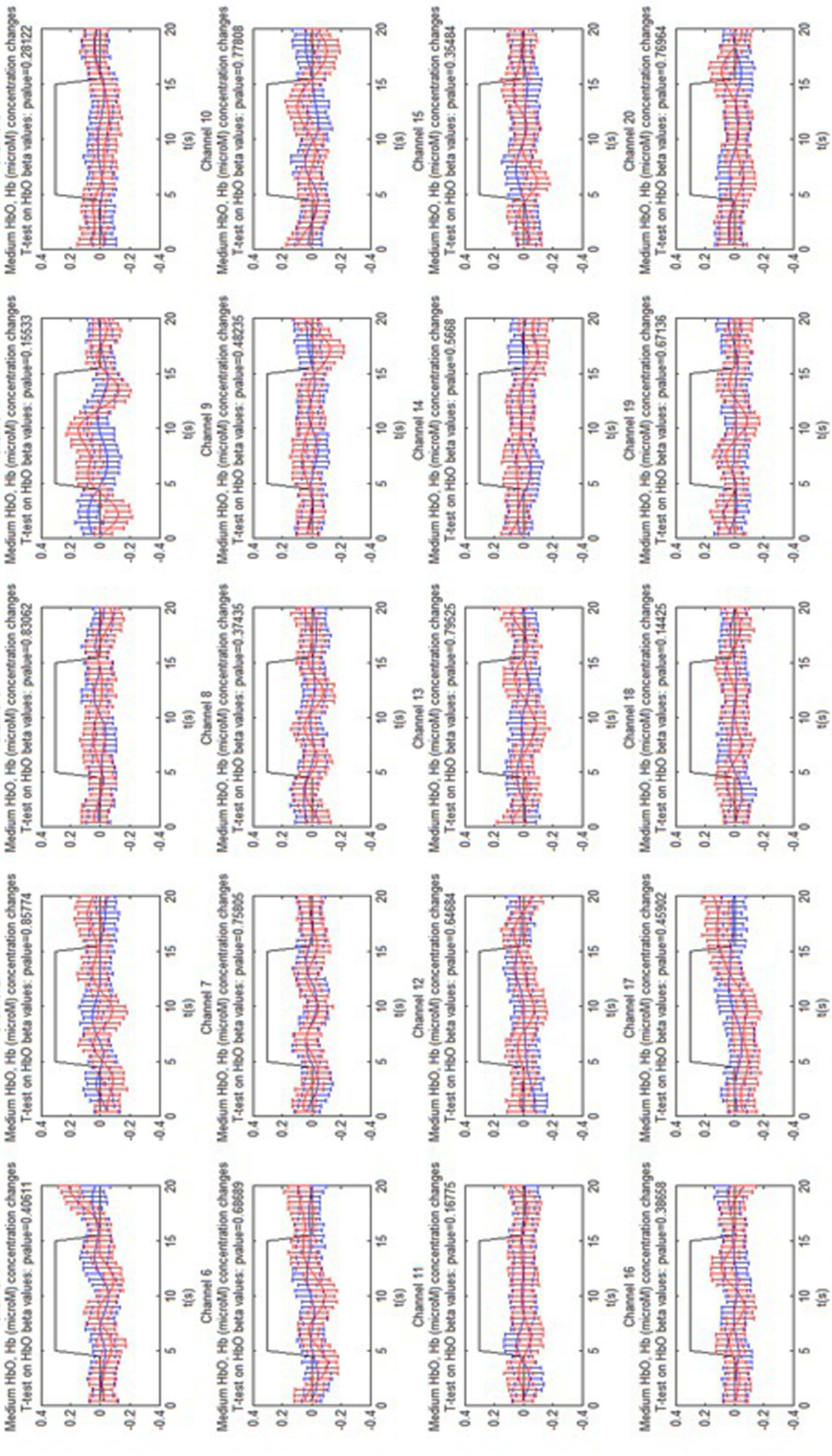

Channel 1
Medium HbO, Hb (microM) concentration changes
T-test on HbO beta values: pvalue=0.40611
Channel 2
T-test on HbO beta values: pvalue=0.85774
Channel 3
T-test on HbO beta values: pvalue=0.83062
Channel 4
T-test on HbO beta values: pvalue=0.15533
Channel 5
T-test on HbO beta values: pvalue=0.28122
Channel 6
T-test on HbO beta values: pvalue=0.68689
Channel 7
T-test on HbO beta values: pvalue=0.75805
Channel 8
T-test on HbO beta values: pvalue=0.37435
Channel 9
T-test on HbO beta values: pvalue=0.48235
Channel 10
T-test on HbO beta values: pvalue=0.77808
Channel 11
T-test on HbO beta values: pvalue=0.16775
Channel 12
T-test on HbO beta values: pvalue=0.64684
Channel 13
T-test on HbO beta values: pvalue=0.79525
Channel 14
T-test on HbO beta values: pvalue=0.5668
Channel 15
T-test on HbO beta values: pvalue=0.35484
Channel 16
T-test on HbO beta values: pvalue=0.38658
Channel 17
T-test on HbO beta values: pvalue=0.45902
Channel 18
T-test on HbO beta values: pvalue=0.14425
Channel 19
T-test on HbO beta values: pvalue=0.67136
Channel 20
T-test on HbO beta values: pvalue=0.76964
t(s)

Supplementary Figure Legends

Figure S1: Average time course changes in HbO_2 and HHb concentration (µM), ±SEM (Standard Error of the Mean), for each source–detector couple, in response to H-F stimulus.

Figure S2: Average time course changes in HbO_2 and HHb concentration (µM) for each source–detector couple, in response to M stimulus in Experiment 1.

Figure S3: Average time course changes in HbO_2 and HHb concentration (µM), ±SEM (Standard Error of the Mean), for each source–detector couple, in response to H-A stimulus.

Figure S4: Average time course changes in HbO_2 and HHb concentration (µM) for each source–detector couple, in response to M stimulus in Experiment 2.
